# Comparative main effects, mediators, and moderators of cognitive behavioral therapy, acceptance and commitment therapy, and emotional awareness and expression therapy for chronic spinal pain: Randomized controlled trial rationale and protocol

**DOI:** 10.1016/j.conctc.2025.101428

**Published:** 2025-01-06

**Authors:** John W. Burns, Mark A. Lumley, Kevin E. Vowles, Mark P. Jensen, Melissa A. Day, Howard Schubiner, Emma Jaszczak, Britney Abro, Sarah H. Addicks, Michael J. Bordieri, Michael M. Dow, Shoshana Krohner, Zyanya Mendoza, Eric C. Meyer, Danielle Z. Miro, Hallie Tankha, David S. Tubman, Jolin B. Yamin, Dokyoung S. You

**Affiliations:** a Department of Psychiatry and Behavioral Sciences, Rush University Medical Center, Chicago, IL, USA; b Department of Psychology, Wayne State University, Detroit, MI, USA; c School of Psychology, Queen's University Belfast, Belfast & Centre for Pain Rehabilitation, Belfast Health and Social Care Trust, Northern Ireland, UK; d Department of Rehabilitation Medicine, University of Washington, Seattle, WA, USA; e School of Psychology, The University of Queensland, Brisbane, Queensland, Australia; f College of Human Medicine, Michigan State University, East Lansing, MI, USA; g School of Health, Augsburg University, Minneapolis, MN, USA; h Department of Psychology, Murray State University, Murray, KY, USA; i Private Practice, Longmont, CO, USA; j Department of Psychiatry, Montefiore/Albert Einstein College of Medicine, New York, NY, USA; k Z Psychology Inc., Greater Los Angeles, California, USA; l Department of Counseling and Behavioral Health, University of Pittsburgh, Pittsburgh, PA, USA; m Private Practice, Reno, NV, USA; n Department of Wellness and Preventive Medicine, Cleveland Clinic, Cleveland, OH, USA; o Private Practice, Dayton, OH, USA; p Brigham and Women's Hospital, Harvard Medical School, Boston, MA, USA; q Department of Anesthesiology, Perioperative, and Pain Medicine, Stanford University, Palo Alto, CA, USA

**Keywords:** Chronic pain, Psychological therapy, Mechanism, Mediator, Moderator

## Abstract

**Background:**

Chronic spinal (back/neck) pain is common and costly. Psychosocial treatments are available but have modest effects. Knowledge of treatment mechanisms (mediators and moderators) can be used to enhance efficacy. Trials that directly compare different treatments are needed to determine which mechanisms are treatment-specific, which are shared across treatments, and which contribute the most to outcomes.

**Methods:**

We will conduct a 4-arm randomized, controlled clinical trial to compare the main effects, mediators, and moderators of three pain therapies: Cognitive-Behavioral Therapy, Acceptance and Commitment Therapy, and Emotional Awareness and Expression Therapy in adults with chronic spinal pain. Following baseline assessment of outcomes variables (two primary outcomes: pain intensity and pain interference) and potential mediators and moderators, we will randomize participants (up to 460) to one of the treatments or usual care control. Treatments will be conducted individually each week for 8 weeks via telehealth. We will conduct weekly assessments of both potential mediators and outcomes, as well as post-treatment and 6-month follow-up assessments. We will test whether any of the therapies is superior to the others (Aim 1); identify mediators that are specific to treatments and those that are shared across treatments (Aim 2); and identify baseline moderators that are specific to treatments or shared across treatments, and moderated mediators of treatments (Aim 3).

**Discussion:**

The findings from this project can be used to improve the effects of psychosocial chronic pain treatments by identifying the most powerful specific and shared mechanisms and revealing for whom the mediator-outcome pathways are strongest.

## 1 Introduction

Chronic spinal or axial (back, neck) pain is a major public health concern [1,2]. Various psychosocial therapies show evidence of reducing pain and/or improving behavioral and emotional functioning, but these treatments have been shown to have modest effects on average [[3], [4], [5]], which is a shortcoming of these otherwise promising approaches. We can boost treatment efficacy of existing treatments, or develop approaches capable of producing enhanced outcomes by identifying the most potent mechanisms of these treatments as well as discover how to better match patients to specific treatments [6]. Our planned clinical trial compares not only the outcomes but also the mechanisms and predictors/moderators of three psychosocial pain therapies: Cognitive Behavioral Therapy (CBT) [7]; Acceptance and Commitment Therapy (ACT) [8]; and Emotional Awareness and Expression Therapy (EAET) [9].

### 1.1 A strategy to increase efficacy: the study of mechanisms

One strategy to enhance the effects of interventions is to increase understanding of treatment mechanisms, by identifying the nature and effects of both mediators and moderators [10]. That is, to boost pain treatment effects, we need to know by what processes, in general, treatments work and for exactly whom, in particular, the processes are most strongly activated.

*Mediators*. Implicit in many psychosocial pain treatment theories is the assumption that different treatments affect outcomes via mediators that are unique or specific to that treatment. Few studies, however, have directly compared different treatments regarding their mediators, and the limited evidence suggests that different pain treatments may operate mostly (but perhaps not entirely) via shared or general (rather than treatment-specific) mediators [11]. Shared mediators include, for example, changes in pain-specific cognitions, emotions, and behaviors [[12], [13], [14]], as well as general psychotherapy mediators such as positive outcome expectancies and the therapeutic alliance [11,15].

*Moderators*. Focusing solely on group averages misses information reflected in variability in treatment response—individuals respond with different levels of improvement [16]. The search for treatment moderators, however, has had limited success, owing to at least five factors: (a) most research has been conducted on samples too small to support moderator analyses; (b) most analyses to date have been conducted post-hoc, exploring variables that happen to be available rather than following *a priori* theory and hypotheses; (c) moderator research rarely compares different treatments; (d) the identification of moderators has often proceeded independently of understanding treatment mediators; and (e) research often finds non-specific “predictors” of treatment outcomes; that is, baseline characteristics that predict outcomes similarly for multiple treatments [17]. This trial addresses these limitations, including the use of a theoretical model to guide the identification of potential moderators (and moderated mediation pathways) of psychological interventions for pain: the Limit, Activate, and Enhance (LA&E) Model [18]. This model supports theory-based predictions regarding moderators as a function of whether treatments limit maladaptive responses, activate adaptive responses, and enhance treatment outcomes based on patient strengths and resources.

### 1.2 New directions and the present study

To test the presence of mechanisms (i.e., mediators and moderators)—whether they are specific to or shared across treatments—one must conduct large trials of multiple, conceptually and technically different treatments [19,20]. In this trial, individuals with chronic spinal pain will be randomized to CBT, ACT, EAET, or treatment as usual (TAU). The three treatments were chosen because of evidence of efficacy and because they are based on divergent theories, propose different mechanisms of action, and use different techniques. The TAU control is needed to determine whether any specific/shared mechanisms are just artifacts of changes that naturally occur over time or with participation in a study.

Each treatment will consist of 8 weekly sessions; potential moderators will be assessed at baseline, and mediator and outcome variables will be assessed at baseline, weekly throughout treatment (or TAU), and at 6-month follow-up. Pain intensity and interference are co-primary outcomes, and post-treatment is the primary endpoint. We will compare the main effects of the three therapies with each other and TAU, but more importantly, we will characterize mediator, moderator, and mediated-moderator effects over the course of therapies. Doing so will clarify which mediators change across different treatments, which predict outcomes, and which are moderated by baseline characteristics.

### 1.3 Specific aims

*Aim 1: Main effects*. The literature usually finds few or no outcome differences among therapies; thus, we hypothesize that the three treatments will result in better outcomes than TAU but not differ in outcomes among themselves, although EAET is newer, and initial tests suggest possible better outcomes than CBT in some populations [[21], [22], [23]] (Table 1 presents the full list of primary and secondary outcomes as well as possible mediators and moderators.).

**Table 1:** Measures in the trial and schedule of administration.

Measure	Screen	Baseline	Week/sessions 1 to 7	Post-tx (week 8: TAU	6-month follow-up
On-line screen items	X				
Inclusion/exclusion criteria (Zoom interview)	X				

**PRIMARY OUTCOMES: ALL TIME POINTS**					
BPI-4 (4-item Pain severity scale)		X	X	X	X
PROMIS Pain Interference SF 8a		X	X	X	X

**SECONDARY OUTCOMES: ALL TIME POINTS**					
Physical function: PROMIS 4-item (from PROMIS-29)		X	X	X	X
Anxiety: PROMIS 4-item (from PROMIS-29)		X	X	X	X
Depression: PROMIS 4-item (from PROMIS-29)		X	X	X	X
Depression: PHQ-8		X	X	X	X
Fatigue: PROMIS 4-item (from PROMIS-29)		X	X	X	X
Sleep disturbance: PROMIS 4-item (from PROMIS-29)		X	X	X	X
Anger: PROMIS Emotional distress-Anger 5a		X	X	X	X
Positive affect (PANAS-SF-5-item)		X	X	X	X
PCL-5 Short Form (4 items)		X	X	X	X
Pain Stages of Change Questionnaire (13 Prep/Action items)		X	X	X	X
Opioid Use (past 7 days)		X	X	X	X
Patient Global Impression of Change			X	X	X
Post-treatment Satisfaction Questionnaire (Therapy arms only)				X	X

**POTENTIAL MEDIATORS AND THEORETICALLY-SPECIFIED TREATMENT: ALL TIME POINTS**					
Pain Self-efficacy Questionnaire 4-item (McWilliams); CBT		X	X	X	X
SOPA-2 (Pain control, Disability, Harm, Emotion scales); CBT		X	X	X	X
Behavioral Activation for Depression Scale-Short form; CBT		X	X	X	X
Self-compassion scale (12 items); ACT		X	X	X	X
Chronic Pain Acceptance Questionnaire (8 items); ACT		X	X	X	X
Chronic Pain Values Inventory (6 x 2 items); ACT		X	X	X	X
Emotional Approach Coping Scale (4 items); EAET		X	X	X	X
Emotional Breakthrough Inventory (6 items); EAET		X	X	X	X
Psychological Insight Questionnaire (6 items); EAET		X	X	X	X
Brain and Stress Attributions for Pain (7 items); EAET		X	X	X	X
Pain Anxiety Symptom Scale (4 items); ALL		X	X	X	X
Pain Catastrophizing Scale 6-item (McWilliams); ALL		X	X	X	X

**POTENTIAL MEDIATORS: DURING TREATMENT ONLY**					
Adverse Events			X	X	X
Working Alliance Inventory-Patient (6 items)			X	X	
Working Alliance Inventory- Therapist (6 items)			X	X	
Therapist Checklist of Patient Engagement and Homework			X	X	
Treatment Expectancy and Credibility Questionnaire (Borkovec 1972; from MATCH)			X	X	

**BACKGROUND/MODERATOR ONLY MEASURES**					
Demographic/medical history questions		X			
Chronic Overlapping Pain Conditions		X			
Medication		X			
Trauma History Questionnaire-Modified (25 items)		X			
Big-5 Inventory (25 items; Johns)		X			
Multidimensional Scale of Perceived Social Support (12 items)		X			
Toronto Alexithymia Scale-20		X			
Pain Detect (7 items)		X			
General Pain Treatment Expectancies		X			

**MODERATOR/OUTCOMES (NON-WEEKLY CHANGES)**					
Levels of Emotional Awareness Scale (Forms A then B)		X		X	
Chronic Pain Attributions Scale-Short Form		X		X	X
Other Pain Treatments and Health Care Use		X		X	X
Inventory of Interpersonal Problems-32		X		X	X
ACR Fibromyalgia Diagnostic Criteria 2011		X		X	X
PSYFlex (Psychological Flexibility Scale; 6 items)		X		X	X
TAPS Tool Part II (3-month window)		X		X	X
PCL-5 (PTSD Checklist, 20 items)		X		X	X
Employment status		X		X	X

*Aim 2: Mediators*. Hypothesized treatment-specific mediators (CBT: behavioral activation and pain control beliefs; ACT: pain acceptance and self-compassion; EAET: emotional approach coping and attributions of pain to the brain rather than body) would change primarily in the targeted treatment; and lagged analyses would show that previous-week mediator changes predict next-week outcome changes primarily in the relevant treatment. In contrast, shared (rather than treatment-specific) mediators would show change irrespective of treatment condition, and earlier mediator changes would predict later changes in outcomes across multiple treatments. Analyses will determine which shared mediators are most critical for outcomes.

*Aim 3: Moderators*. We expect that people high in pain catastrophizing will respond best to CBT; those low in catastrophizing and/or high in experiential avoidance will respond best to ACT; and those low in alexithymia and/or with trauma history will respond best to EAET.

*Aim 4: Moderated mediators*. According to the LA&E model, individual differences among participants at baseline (i.e., potential moderators) exert their effects on treatment outcomes because they activate or influence specific mediators within a treatment. For example, a mediator specific to EAET – increases in emotional approach coping – may be activated most robustly among people with a history of trauma (acting here as a moderator) thereby driving favorable outcomes for this subgroup of participants. That is, we will test whether moderators exert effects on outcomes among people in specific treatment conditions via hypothesized mediators.

## 2 Methods

### 2.1 Participants

We aim to randomize 460 adults, recruited from the community and clinics using a range of methods (e.g., flyers in clinics, postings to social media, advertisement in patient advocacy groups, medical center call waiting announcements, ResearchMatch). Participants will report spinal pain—anywhere in the back or neck—as their most prominent pain problem. For inclusion, pain must: (a) be at least 3 months’ duration and; (b) occur at least half the days in the past 6 months; and (c) have both self-reported intensity and interference ratings (past month) of at least 3 (0–10 scale). Additional inclusion criteria are: (d) fluency in English; (e) access to personal computer/tablet; (f) ability to attend weekly telehealth sessions; (g) willingness to be randomized; and (h) residence in a U.S. state participating in psychologist licensure reciprocity (i.e., PSYPACT state).

Exclusion criteria are few so that we can recruit a diverse and generalizable sample. They include: (a) pain thought to be secondary to a disease process (e.g., cancer, autoimmune disease) or neuropathy (e.g., leg pain greater than back pain); (b) alcohol/substance dependency or use disorder within past 2 years; (c) psychotic or bipolar disorder anticipated to interfere with therapy; (d) suicidality; (e) cognitive impairment (2+ errors on 6-item screener [24]); (f) current or past year application for disability or pain-related litigation; or (g) major surgery planned for the next 9 months.

### 2.2 Procedure

The trial was approved by the Rush University Medical Center IRB (other sites relegated authority to Rush) and registered on ClinicalTrials.gov (NCT06044649). All study activities will be conducted remotely: recruitment, screening, and other communication will use email, text, and Zoom; assessments will use REDCap, and telehealth interventions will use Zoom.

**Fig. 1:**
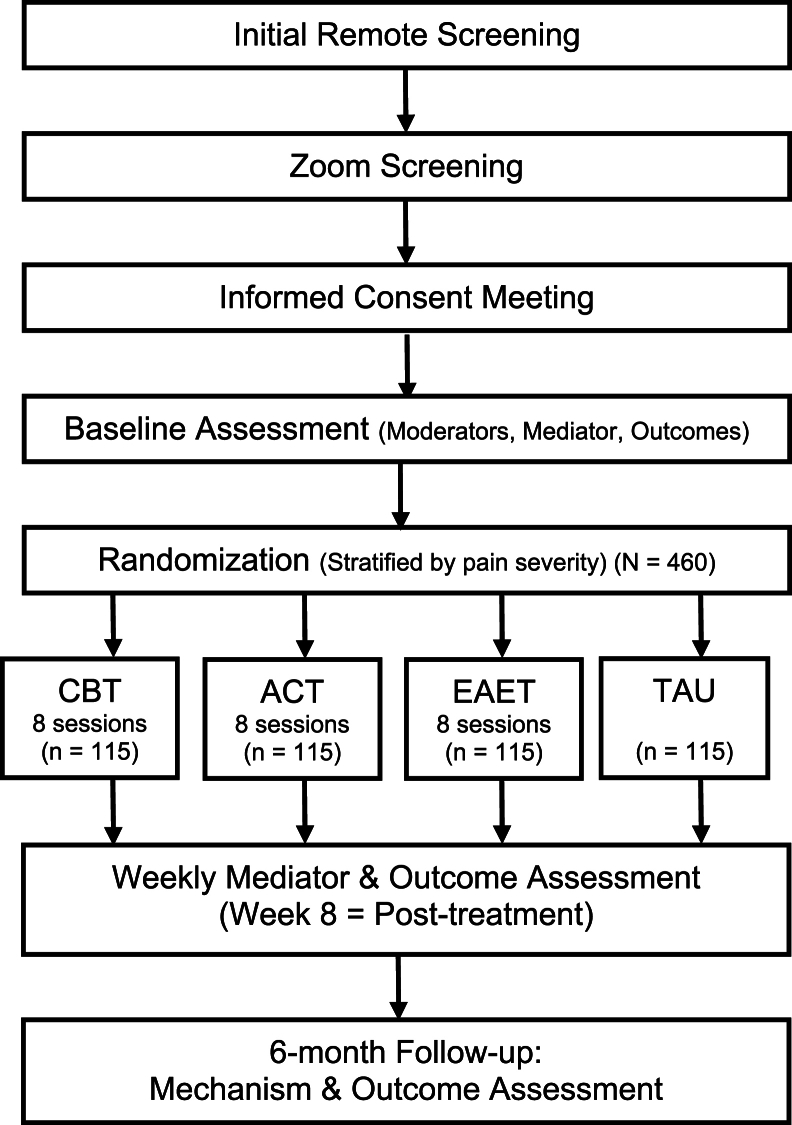
Design of clinical trial.

Fig. 1 presents the design of the trial as a flow chart.

*Screening.* Potential participants will complete a brief survey on-line; those passing this screen will have a Zoom interview to determine eligibility. Staff will describe the study to those who are eligible; if they remain interested, they will receive the consent form and schedule a subsequent consent meeting, at which staff will obtain consent. After consent, participants will receive a REDCap link to baseline (pre-treatment) measures.

*Randomization.* After completing baseline measures, patients will be randomized to one of four conditions. Before the trial, the statistician will generate (with a customized randomization algoritm) randomization assignments, stratified by pain severity (two strata, defined by the average of screening pain intensity and interference ratings: “high” is > 6, “low is ≤ 6); such stratification will increase the likelihood of equivalence among treatment conditions at baseline on these key outcomes. Randomization will also be done in blocks, thus increasing the likelihood that the four arms will have equivalent sample sizes. The sealed randomization schedule will be accessed by a researcher who has no participant contact, who will inform the research assistant of the condition assignment. The assistant will then inform the participant that they are assigned to receive a treatment (but not which one) and that they should schedule Session 1 with a specific therapist, or that the participant should continue their usual care (if assigned to TAU).

*Blinding.* This trial uses several types of blinding. First, participants and study personnel are blinded to condition until after baseline assessment. Then, staff will send a standardized email to participants (copying the therapist, who is assigned based on availability) informing participants of their therapist (or to continue treatment as usual). Participants learn in Session 1 to which therapy they are assigned; a process which should minimize attrition or other biases that can occur when participants are informed about their treatment prior to meeting their therapists. We will also assess participants’ perceived treatment credibility after session 1, at which time participants learn of their assigned therapy and its rationale, This measure can inform the success of blinding and the bias of differential expectations. Subsequently, participants are sent standardized emails for all weekly, post-treatment, and follow-up assessments, which are conducted via REDCap. There are no personal/unstructured interactions between staff and participants that could bias responses; thus, outcome assessment is essentially blinded/unbiased. The study statistician will analyze the data blind to condition assignment.

### 2.3 Treatment conditions

All three treatments (CBT, ACT, and EAET) will be equal in format and duration: 8 sessions (allowed to occur over a 10-week period), 60 min long, held weekly (no closer than 4 days apart), conducted individually and remotely by a study therapist. Each therapy is guided by its own manual, which describes principles and techniques, and includes a patient workbook. This workbook is introduced to the participant in session 1 by the therapist, who will email it to the participant after session 1.

*Cognitive Behavioral Therapy*. CBT's overarching premise is that chronic pain leads to learned, maladaptive patterns of thinking, behaving, and feeling, which exacerbate pain intensity, dysfunction, and distress. The version of CBT used in this study is based on several empirically-supported protocols [[25], [26], [27]]. Chronic pain can be successfully managed by teaching people self-regulation, including: (a) cognitive restructuring of maladaptive pain-related thoughts; (b) relaxation training and reassuring self-statements to reduce emotional distress and arousal; (c) activity-rest cycling to address excessive pain behaviors (e.g., downtime); (d) pleasant activity scheduling to increase the level and range of daily reinforcing activities; (e) problem-solving to identify barriers and develop solutions to cognitive and behavior changes; and (f) a written maintenance plan with short- and long-term goals for applying these skills and dealing with possible setbacks. Weekly homework focuses on practicing these skills.

*Acceptance and Commitment Therapy.* ACT seeks to decrease maladaptive avoidant behavioral responses to chronic pain, enhance engagement in personally-valued activities, and decrease pain-related restrictions. This version of ACT is based on protocols by Vowles [28], which have been successfully used in previous trials [[29], [30], [31]]. ACT helps people identify areas of meaningful functioning that have been adversely impacted by pain, learn methods to enhance pain acceptance in the service of these meaningful areas, and practice present-focused awareness/mindfulness skills to aid in taking effective action towards values and in a manner that is sustainable over the longer term. Sessions include discussions of the impact of pain and distress avoidance efforts, identification of alternatives to this avoidance, establishment of plans for behavior change enabling progress towards valued living, behavior change and experiential exercises (e.g., therapist demonstration; role-playing), and homework to facilitate adaptive behavior change and aid in generalization.

*Emotional Awareness and Expression Therapy.* EAET is based on contemporary pain neuroscience which views chronic pain (especially primary pain) as a brain-based, predictive processing “alarm” system that is unnecessarily activated when the person perceives threat or danger—to either their physical body or their interpersonal and psychological safety. Fearful (but incorrect) beliefs that pain reflects bodily damage as well as fear and avoidance of emotions related to adverse life experiences, trauma, and relationship conflicts can generate, maintain, or exacerbate pain [[32], [33], [34], [35], [36]]. The goal of EAET, therefore, is to greatly reduce or eliminate pain by having people unlearn these fears. This version of EAET is an adapted version of the manual written for group-based treatment of fibromyalgia [23,37]. EAET: (a) educates patients about pain neuroscience and the brain's role in generating pain; (b) uses pain provocation techniques and healthy affirmations to decrease the brain's pain alarm related to perceived bodily injury or damage; (c) facilitates disclosure of stressful or conflictual experiences and emotions; (d) processes emotions related to unresolved traumas and or conflict (i.e., identify, experience, express, reflect on, and release emotions), particularly emotions related to power and autonomy (e.g., anger), connection (tenderness, love), grief (sadness), and self-compassion; and (e) encourages healthy communication with others. Weekly homework exercises include engaging in reading or watching videos, exploring stressors and emotions, and practicing new ways of communicating.

*Treatment as usual.* Participants assigned to TAU will not receive any of the three psychological treatments offered in this study. They will, however, be asked to continue ongoing treatments for pain, including medications and other therapies, as they and their care providers deem appropriate. Similarly, participants assigned to all three treatment conditions will be allowed to continue their usual care.

### 2.4 Therapist training, supervision, fidelity, and adherence

Interventions will be delivered via Zoom by doctoral-level (PhD or PsyD), licensed clinical psychologists who will be “nested within” treatment rather than “crossed over” treatments; that is, a minimum of three psychologists with skill in and commitment to each therapy will conduct it, rather than delivering all three therapies. Conducting only the therapy in which one has expertise is most ecologically valid and reduces concern about bias (diffusion, comparative motivation) that can occur when the same therapist tries to conduct—with confidence, competence, and conviction—three quite disparate treatments, especially those with contrasting views of the possibility and desirability of pain reversibility (EAET and ACT). The trainers/supervisors for the three therapies are Burns (CBT), Vowles (ACT), and Lumley and Schubiner (EAET), each of whom has expertise and a history of training professionals in their therapy. The use of separate supervision/training experts and therapists for each intervention maximizes study “equipoise.” Also, the three lead investigators are committed to the success of each of their therapies; such “adversarial collaboration” reduces concerns about the bias that occurs with investigator allegiance effects.

All study therapists will receive training consisting of remote, 2-day courses. Treatment sessions will be videorecorded (via Zoom) and downloaded/saved for supervision, fidelity assessment, and later process analyses. At the trial's start, weekly supervision will be conducted by the investigator leading each treatment, who will watch all sessions of each therapist's first participant to confirm the therapist's competence and adherence. Observation of additional cases and further supervision/training will be conducted to maintain skills, as needed.

Ongoing verification of therapist adherence to the treatment protocols will be conducted during the trial by clinical psychology doctoral students, who will review the 8-session course of therapy for 20 % of cases from each therapy and therapist, rating the presence of the core components of each therapy. The full course of each therapy will be the target of fidelity ratings because ACT and EAET do not prescribe specific techniques to be conducted during specific sessions - unlike CBT - but rather follow principles that allow the delivery of techniques, as needed by individual patients, in various sessions. The percentage of components presented will be calculated for each therapy, and feedback from coders will be provided to supervisors and therapists to help prevent drift.

### 2.5 Data collection and measures

Table 1 presents all measures and assessment points: baseline, weekly during treatment/usual care, post-treatment (the week 8 assessment), and 6-month follow-up (6 months after post-treatment). The measures are divided into: (a) outcomes (primary and secondary), which are assessed at all time points; (b) mediators, which are assessed at all time points; (c) moderators, which are assessed only at baseline; and (d) another set of potential moderators/secondary outcomes that we assess only at the three major time points (baseline, post-treatment, and 6-month follow-up. Finally, there are several measures that apply only to participants engaged in one of the three interventions (but not TAU), and these will be assessed after each session of treatment. Study therapists will also complete several measures after each session.

This trial has two co-primary outcomes – pain intensity (assessed with the Brief Pain Inventory [38]) and pain interference (PROMIS Pain Interference scale [39]) – because the three therapies have different primary goals: ACT seeks to decrease pain interference, EAET seeks to reduce pain intensity, and CBT targets both outcomes. Our primary study endpoint for main effects is post-treatment. All measures included in this trial have evidence supporting their psychometric qualities. Most measures are participant self-reports, although therapists provide ratings after each session. Because there are numerous patient-report measures, we will use brief measures or short forms to minimize respondent burden, especially for the weekly assessments.

Within 48 h of each treatment session (or weekly for TAU), participants will complete the online survey (REDCap), which contains the battery of study outcomes and mediators. The data collected after session 8/week 8 will be considered the “post-treatment” assessment. Participants will have the follow-up assessment 6 months later. Treatment completion is defined as completing at least 6 of the 8 planned sessions. Participants who do not complete treatment will be encouraged to provide post-treatment and follow-up assessments. Participants will be paid $50 for each of the baseline, post-treatment, and follow-up assessments, and $25 for each of the 7 weekly assessments, for a possible total of $325; treatment is provided for free.

### 2.6 Adverse events

To ensure participant safety, we will monitor for adverse events via patient self-report on the weekly surveys and by therapist report. Adverse events are defined as any unfavorable or unintended symptom, sign, or disease that may or may not be related to the treatment or study participation. We will also monitor for serious adverse events (i.e., death, hospitalization, emergency room visits, suicide plans or attempts) at each treatment session. The funding agency (National Institute of Nursing Research) has approved our data safety and monitoring plan, which calls for a person independent of the study and the study institutions to serve as a Safety Officer. A full Data Safety Monitoring Board will not be convened. We will consult our Safety Officer within 24 h upon discovery of any serious adverse event (whether or not it appears to be study related) as well as any non-serious adverse events that are judged by the participant or therapist as definitely or possibly study-related. Such events may be reported to the IRB and/or NIH as determined by the Safety Officer.

### 2.7 Power and statistical analyses

Simulation-based power analyses were conducted to justify the sample size based on linear mixed model (LMM) analyses. We powered for the fixed effects of treatment condition and interaction with time to ensure power of at least 80 % at the 2-sided type I error rate of *p* = 0.05, and assuming attrition of approximately 15 % (based on our prior trials) that is evenly spaced throughout program timepoints. This analysis revealed that a sample size of 460 randomized participants should provide greater than 90 % power to detect differences among treatment conditions (e.g., group × time interactions), provided endpoint group differences in outcomes that are of clinical significance (i.e., d = 0.4–0.5 or higher). Analysis of sample size for models using mediators as lagged time-varying covariates was conducted based on simulations [40,41], which suggested that power nearly always exceeded 0.90 to detect outcome changes across time, dependent on change in mediators, with a sample size of 460 and number of measurements exceeding eight.

Primary analyses include all randomized participants (intent-to-treat). Analysis of treatment condition differences across treatment and follow-up time points will use maximum likelihood-based LMM for each outcome [42]. Models will be adjusted for covariates as appropriate. To test for main effects (Aim 1), models will compare the four conditions to detect differential slopes of change from baseline to post-treatment on the two primary outcomes and secondary outcomes. Significant omnibus (4-arm) tests will be followed by post-hoc comparisons of each treatment with TAU as well as each treatment with each other. Similar analyses will compare treatment conditions from baseline to 6-month follow-up.

We will test for mediators (Aim 2) that are, in theory, specific to each treatment, as well as for shared mediators, which operate across all treatments. Evidence of *specific* mediators will be revealed by the following (a) putative specific mediators (e.g., pain acceptance in ACT): will show the largest pre-post changes in the relevant treatment condition (e.g., ACT pain acceptance changes > CBT, EAET, TAU) (b) mediators NOT specific to a treatment (e.g., pain acceptance in CBT); will NOT change substantially in that treatment; (c) in lagged analyses, substantial changes in the mediators will precede and predict substantial subsequent changes in outcomes. Evidence of *shared* mediators will be revealed by the following: (a) mediators hypothesized as specific to a treatment as well as mediators thought NOT to be specific to a treatment will show substantial treatment changes in that treatment; (b) in lagged analyses, substantial changes in mediators specific to and not specific to a treatment, and general mediators (e.g., therapeutic alliance) will precede and predict substantial subsequent changes in outcomes; and (c) changes in mediators specific to a treatment and changes in mediators not specific to a treatment (i.e., shared mediators) will predict unique variance in outcome changes.

Baseline characteristics hypothesized to serve as moderators on outcomes will be examined across all four conditions (Aim 3). Evidence of a moderator will be revealed by the following: (a) a significant Treatment Condition x Moderator × Time interaction for weekly changes in an outcome; (b) Moderator x Time simple effects such that the moderator is related significantly to outcome change in one or two but not three treatment conditions. Significant interaction terms would indicate that differences in outcome across treatment conditions exist based on these moderating factors.

Finally, we will examine whether associations between lagged mediator changes and outcome changes over the course of treatment depend on hypothesized moderators; that is, moderated-mediator effects (Aim 4). This approach addresses whether specific week-to-week lagged relationships between mediator and outcome changes depend on levels of baseline characteristics.

### 2.8 Trial status

The trial, funded with a 4-year NIH grant (originally planned as a 5-year grant) began screening participants in November 2023 and randomized its first participant on December 7, 2023. It is expected that randomization will continue through spring 2026, with follow-up and analyses occurring in the subsequent year.

## 3 Discussion

This RCT is innovative and can expand knowledge of psychosocial treatments in several ways. First, results will clarify what makes psychosocial pain treatments work. There is much value in examining whether theorized mechanisms (mediators and moderators) are indeed actual mechanisms, whether certain treatments exert effects via mechanisms that are specific to a treatment, or whether certain mechanisms operate across different treatments. Such findings will reveal which one or more mediators are driving change within each treatment, and for whom, and whether other putative mediators may need to be targeted to a greater degree. Second, RCTs comparing different pain interventions are very rare, but this trial compares three conceptually- and technically-unique therapies, which have never been compared with each other, and the trial does so with experimental equipoise. Such a comparison will not only determine whether one therapy is more efficacious than another, but importantly, test whether mediators and moderators are specific or generalized and which should be targeted to enhance treatment effects. Third, this trial will examine general mechanisms common to many efficacious interventions (e.g., the therapeutic alliance). Such findings could strengthen efforts to enhance these factors in pain treatments and direct training that not only focuses on techniques but reinforces the importance of, for instance, establishing and maintaining sound therapeutic relationships or expectations. Finally, this trial will be conducted entirely remotely. Almost all prior intervention research has been conducted in-person, but there are many limitations of that approach, including poor access for those living far from treatment centers, who have limited transportation or time, or whose health problems or caregiver duties prevent participation. This trial will provide data to compare with prior in-person research while simultaneously leading the field into the next generation of treatment delivery research and practice.

This trial has several potential limitations or challenges. First, the choice of control conditions for comparative RCTs is controversial. We include TAU as an ecologically-valid comparison because “usual care”—without specialized additional psychological treatment—is what most patients actually experience. Also, active control conditions such as support or education, although controlling for non-specific factors, may be efficacious themselves [43], thereby confounding comparisons that seek to isolate the active ingredients of target treatments. Second, studies vary in their targeted chronic pain problem; some are very heterogeneous (e.g., any musculoskeletal pain), impeding attempts to match treatments to specific pain conditions, whereas others (e.g., low back pain) seem homogeneous but ignore the fact that many patients have other pain locations, commonly in the spinal/axial region. Thus, we will target patients with spinal pain but also assess the extent of musculoskeletal pain across the body and test this as a possible moderator. Third, a trial conducted remotely might raise concerns about equitable access due to technological differences; however, we think that access and retention will be at least as good, and likely better than occurs in trials that require in-person attendance [44]. Although we do not know how these interventions delivered via telehealth will compare to the larger literature generated via face-to-face interventions, we think it is vital to obtain such remotely-delivered intervention data. Finally, because study participants may engage in other psychosocial or medical pain treatments during the trial, we will assess for their presence and test this factor either as a moderator or covariate.

This trial has implications for policy and practice. Results will show which mechanisms should be activated to bring about treatment success, and conversely, which mechanisms are less important than previously thought. Providers will have empirical bases for favoring certain techniques and change processes and for de-emphasizing others. Results will support new guidelines regarding patient characteristics contributing to the best treatment matching, so that providers will gain empirically-supported procedures for screening patients and recommending participation in treatments best suited to them.

## 4 Disclosures

Mark A. Lumley is a paid research consultant to CognifiSense, Inc, which develops virtual reality interventions for chronic pain. He also facilitates workshops to train clinicians in pain treatments and sometimes receives fees for doing so.

Kevin E. Vowles provides paid chronic pain treatment training and supervision for organizations that provide treatment services for chronic pain disorders, including Kaiser Permanente Southern California (USA) and Connect Health Ltd (UK).

Mark P. Jensen has written or edited several books on the use of an intervention that can be used for chronic pain management (therapeutic hypnosis) and received royalties from the sales of these books. He also facilitates workshops to train clinicians in the use of therapeutic hypnosis for pain management, and sometimes receives fees for doing so. He also owns equity in a company that is developing a digital therapeutic that can provide therapeutic hypnosis.

Howard Schubiner has written or edited several books on chronic pain treatment and received royalties from the sales of these books. He facilitates workshops to train clinicians in chronic pain treatments and sometimes receives fees for doing so. He owns equity in two companies that provide education for patients and clinician training: Freedom From Chronic Pain and Oviddx.com.

Shoshana Krohner facilitates workshops to train clinicians in chronic pain treatments and sometimes receives fees for doing so. She co-owns a company that provides consultation and training to clinicians who treat chronic pain.

No other authors have disclosures.

## CRediT authorship contribution statement

**John W. Burns:** Writing – original draft, Supervision, Project administration, Methodology, Funding acquisition, Conceptualization. **Mark A. Lumley:** Writing – original draft, Supervision, Project administration, Methodology, Funding acquisition. **Kevin E. Vowles:** Writing – review & editing, Supervision, Resources, Project administration, Funding acquisition, Conceptualization. **Mark P. Jensen:** Writing – review & editing, Methodology, Funding acquisition, Conceptualization. **Melissa A. Day:** Writing – review & editing, Methodology, Funding acquisition, Conceptualization. **Howard Schubiner:** Writing – review & editing, Supervision, Methodology, Funding acquisition, Conceptualization. **Emma Jaszczak:** Writing – review & editing, Project administration, Methodology, Data curation. **Britney Abro:** Writing – review & editing, Project administration, Methodology, Data curation. **Sarah H. Addicks:** Writing – review & editing, Data curation. **Michael J. Bordieri:** Writing – review & editing, Data curation. **Michael M. Dow:** Writing – review & editing, Data curation. **Shoshana Krohner:** Writing – review & editing, Data curation. **Zyanya Mendoza:** Writing – review & editing, Data curation. **Eric C. Meyer:** Writing – review & editing, Data curation. **Danielle Z. Miro:** Writing – review & editing, Data curation. **Hallie Tankha:** Writing – review & editing, Data curation. **David S. Tubman:** Writing – review & editing, Data curation. **Jolin B. Yamin:** Writing – review & editing, Data curation. **Dokyoung S. You:** Writing – review & editing, Data curation.

## Funding source

This trial is made possible by an R01 grant from the NIH/National Institute of Nursing Research: R01 020610; “Comparative Mechanisms (Mediators, Moderators) of Psychosocial Chronic Pain Treatments”; Multiple PI: John Burns and Mark Lumley.

## Declaration of competing interest

The authors declare that they have no known competing financial interests or personal relationships that could have appeared to influence the work reported in this paper.

## Data Availability

No data was used for the research described in the article.
